# Does the age of packed red blood cells, donor sex or sex mismatch affect the sublingual microcirculation in critically ill intensive care unit patients? A secondary interpretation of a retrospective analysis

**DOI:** 10.1007/s10877-022-00877-3

**Published:** 2022-06-03

**Authors:** Demian Knobel, Jonas Scheuzger, Andreas Buser, Alexa Hollinger, Caroline E. Gebhard, Rita Achermann, Anna Zaiser, Yann Bovey, Chiara Nuciforo, Julie Noëmie Netzer, Aline Räber, Jasprit Singh, Martin Siegemund

**Affiliations:** 1 Intensive Care Unit, Department of Acute Medicine, University Hospital Basel, Petersgraben 4, 4031 Basel, Switzerland; 2 Regional Blood Transfusion Service, Swiss Red Cross, Basel and Department of Haematology, University Hospital Basel, Basel, Switzerland; 3 Medical Faculty of the University of Basel, Basel, Switzerland

**Keywords:** Storage lesions, Transfusion, Red blood cell transfusion, Sublingual microcirculation, Oxygen supply, Sex mismatch, Capillary blood flow packed red blood cells, age

## Abstract

In vitro studies have thoroughly documented age-dependent impact of storage lesions in packed red blood cells (pRBC) on erythrocyte oxygen carrying capacity. While studies have examined the effect of pRBC age on patient outcome only few data exist on the microcirculation as their primary site of action. In this secondary analysis we examined the relationship between age of pRBC and changes of microcirculatory flow (MCF) in 54 patients based on data from the Basel Bedside assessment Microcirculation Transfusion Limit study (Ba^2^MiTraL) on effects of pRBC on sublingual MCF. Mean change from pre- to post-transfusion proportion of perfused vessels (∆PPV) was + 8.8% (IQR − 0.5 to 22.5), 5.5% (IQR 0.1 to 10.1), and + 4.7% (IQR − 2.1 to 6.5) after transfusion of fresh (≤ 14 days old), medium (15 to 34 days old), and old (≥ 35 days old) pRBC, respectively. Values for the microcirculatory flow index (MFI) were + 0.22 (IQR − 0.1 to 0.6), + 0.22 (IQR 0.0 to 0.3), and + 0.06 (IQR − 0.1 to 0.3) for the fresh, medium, and old pRBC age groups, respectively. Lower ∆PPV and transfusion of older blood correlated with a higher Sequential Organ Failure Assessment (SOFA) score of patients upon admission to the intensive care unit (ICU) (*p* = 0.01). However, regression models showed no overall significant correlation between pRBC age and ∆PPV (*p* = 0.2). Donor or recipient sex had no influence. We detected no significant effect of pRBC on microcirculation. Patients with a higher SOFA score upon ICU admission might experience a negative effect on the ∆PPV after transfusion of older blood.

## Background

Transfusion of packed red blood cells (pRBC) to correct anaemia is a frequent therapy in intensive care unit (ICU) patients and is used against blood loss as well as to improve oxygen delivery [1]. Although considered safe and potentially lifesaving, the risk of adverse transfusion reactions should be acknowledged.

Packed red blood cells may develop so-called storage lesions during their shelf life. The oxygen-carrying capacity of pRBC is lowered by the reduction of adenosine triphosphate (ATP) and 2,3-diphosphoglycerate, membrane phospholipid peroxidation and vesiculation, protein oxidation, loss of deformability, and increased osmotic fragility [2–4]. This leads to increased haemolysis and the occurrence of cell-free haemoglobin [5], which may in turn cause vasoconstriction due to scavenging of nitric oxide (NO) by free haemoglobin [5, 6]. Overall, this may reduce perfusion of the microcirculation [7], resulting in worsened tissue oxygenation [3].

Nowadays, microcirculatory flow (MCF) can be measured sublingually at the bedside using handheld microscopes [8, 9], facilitating detection of the effects of pRBC on MCF and using it as a surrogate marker for organ perfusion. Thus, measuring MCF could be a valuable extension to guide transfusion decisions [10].

In order to evaluate the role of pRBC on sublingual microcirculation at different haemoglobin transfusion thresholds, we conducted a prospective observational trial [10], in which Scheuzger et al. showed that the influence of pRBC on microcirculatory vessel perfusion was independent from the initial haemoglobin (Hb) level. MCF improved in approximately one third of all patients after transfusion of pRBC. This improvement was inversely correlated with pre-transfusion values. Patients with an initial proportion of perfused vessels (PPV) of 88% or lower improved their MCF after transfusion of one pRBC.

In this secondary analysis of this prospective cohort, we aim to test the hypothesis that storage lesions of older pRBC may negatively impact MCF.

## Methods

This retrospective single-centre analysis is based on the observational study of Scheuzger et al. [10] (see Sect. 2.2 below). More details on patient-cohort and methods are listed in this previous trial, which was approved by the local ethics committee (Ethics Committee of Northwest and Central Switzerland, EKNZ, project ID: 2017-01190).

### Patients

Patients were recruited from the intensive care unit (ICU) of the University Hospital Basel, Switzerland, between September 2017 and September 2018. Upon discretion of the treating intensivist sixty-four patients with anaemia (Hb < 90 g/l) in sepsis, after trauma, or with postoperative bleeding receiving pRBC were included. Transfusion threshold (TTH) was set at 75 g/l or 90 g/l in patients with cardiac comorbidities.

Patients aged < 18 years and those requiring mechanical assist devices, presenting with orofacial trauma, active oral bleeding, or any other condition complicating sublingual microcirculatory measurement were excluded.

### Protocol

In the Ba^2^MiTraL study, sublingual microcirculatory measurements were performed within 1 h before (T1) and within 1 h after (T2) transfusion of one unit (300 ml) of a leukocyte-depleted RBC. At each time point, the best three measurements were used for the analysis^.^ For all measurements CytoCam© (Braedius, Netherlands) based on incident dark-field illumination technology was used. The SOFA score was recorded at T1.

In the present analysis, we completed the dataset for the 64 patients of the Ba^2^MiTraL trial with information concerning the age (days) of the pRBC, sex and blood group of donor. We excluded ten patients due to rapid transfusion without follow-up measurement or missing registration numbers of the pRBC. For the evaluation of the effect of sex on chance in transfusion values, only 43 cases were included due to missing information on the donor’s sex.

### MCF assessment

The videos assessed for the Ba^2^MiTraL trial were analysed offline according to De Backer, as recommended by the producer of CytoCam due to the missing possibility of automatic analysis for the proportion of perfused vessels (PPV) for the team. To stabilize the video sequences, CytoCam Tools version 1.7.12 (Braedius, Netherlands) was used [11].

Several possible parameters are available to describe the quality of the MCF. The two most important parameters, the PPV and the microvascular flow index (MFI), were mainly used in our analysis [12, 13].

### Assessment of transfused pRBC

For graphical representation of the change in PPV (∆PPV) after transfusion, age of blood was divided into 3 groups: fresh (8 to 14 days), medium (15 to 34 days), and old blood (35 to 48 days): As relevant changes in erythrocytes have been reported to occur after two weeks [7]. For relatively old blood, the threshold was set at five weeks (≥ 35 days) in accordance with thresholds used in previous studies [7, 14–17]

In our hospital, pRBC are suspended in two different storage solutions: saline-adenine-glucose-mannitol (SAG-M) and phosphate-adenine-glucose-guanosine-saline-mannitol (PAGGS-M:), which allow a storage time of ≤ 42 days and ≤ 49 days, respectively [18].

### Statistical analysis

∆MFI and ∆PPV were calculated by taking the difference between the mean of the three measurements from pre- and post-transfusion values of MFI or PPV, respectively. For the assessment of inter-measurement variance, ANOVA was used. The correlation between the change in ∆PPV and ∆MFI and the age of the pRBC was modelled using linear regression.

To investigate whether the association between blood age and ∆PPV and ∆MFI correlates with the Sequential Organ Failure Assessment (SOFA) score, we divided the patients into a group of critically ill patients with a SOFA score ≥ 10 (n = 18) and patients with a SOFA score < 10 (n = 36). This variable was included as the covariate in the linear regression model as an interaction term with pRBC age.

In addition, pRBC donor’s sex was set in relation to the ∆PPV. In particular, we wanted to examine whether there was a correlation of those delta values in donor-recipient sex-mismatch. Regression models were used to calculate the influence of blood age on the ∆PPV for the two groups.

For all tests, alpha error (*p* < 0.05) was considered significant. Calculations were performed using R Studio©, version 3.6.1 (2019–07-05; R Studio©, Inc., Boston, MA, USA, 2009–2020). For non-normally distributed values, data are presented as median and interquartile range (IQR), otherwise as mean and standard deviation (SD).

## Results

Baseline characteristics are shown in Table 1. Transfusion figures regarding blood groups and donor-recipient sex-mismatches are listed in the Appendix (Appendix Table 5).

**Table 1:** Patient characteristics

Characteristic	Total (*n* = 54)	Fresh (*n* = 9)	Medium (*n* = 27)	Old (*n* = 18)
Male, *n* (%)	30 (55.6)	3 (33.3)	15 (55.6)	12 (66.7)
Mean age, SD (years)	64.9 (15.2)	61.2 (15.0)	69.0 (14.7)	60.6 (15.2)
SOFA score (points)	7 (3–11)	10 (3–12)	7 (4–11)	5 (3–7)
Septic shock, *n* (%)	11 (20.4)	2 (22.2)	6 (22.2)	3 (16.7)
Cardiogenic shock, *n* (%)	16 (29.6)	3 (33.3)	9 (33.3)	4 (22.2)
Haemorrhagic shock, *n* (%)	18 (33.3)	1 (11.1)	7 (25.9)	10 (55.6)
Mechanical ventilation, *n* (%)	24 (44.4)	5 (55.6)	11 (40.7)	8 (44.4)
Hb before pRBC (g/l)	74.5 (72–79)	79 (72–85)	74 (72–78)	74.5 (73–78)
Blood type donor, 0, *n* (%)	20 (37)	4 (7)	10 (19)	6 (11)
Length of ICU stay (days)	5 (2–12)	8 (4–16)	4 (1.5–11)	5 (2.3–11)
Death in ICU, *n* (%)	5 (9.3)	0 (0)	3 (11.1)	2 (11.1)

*SD* standard deviation; *SOFA* sequential organ failure assessment; *Hb* haemoglobin; *pRBC packed* red blood cells; *ICU* intensive care unit

If not mentioned differently, all values above are medians with corresponding interquartile range (IQR)

Thirty patients were male (55.6%), and mean age at time of transfusion was 64.9 years (SD = 15.2). Median time of processing and storage of pRBC from donation to transfusion was 28.5 days. Maximum time of storage was 42 days, except for one patient who received 48 day-old pRBC, which was suspended in PAGGS-M.

Median change from pre- to post-transfusion for PPV was + 3.45% (IQR − 1.6 to 10.7), and mean change for MFI was + 0.17 (SD = 0.38). The variance in measurements within subjects were 18.6% for PPV pre-transfusion, 23.2% for PPV post-transfusion, 10.8% for MFI pre-transfusion and 19.5% for MFI post-transfusion. Table 2 displays the results of the regression analysis for the overall correlation between the change in PPV from pre- to post-transfusion (∆PPV) and the age of the pRBC, both of which were not statistically significant (*p* = 0.29).

**Table 2:** Regression model of ∆PPV and age of pRBC

Co-variable	Estimate	Confidence interval	p-value
Intercept ∆PPV	10.41	(1.02 to 19.81)	0.03
Age of pRBC	− 0.17	(− 0.49 to 0.15)	0.29

Adjusted R^2^: 1.4%; *n* = 54

*∆*Difference between pre- and post-transfusion measurement

*PPV* proportion of perfused vessels; *pRBC* packed red blood cells

**Fig. 1:**
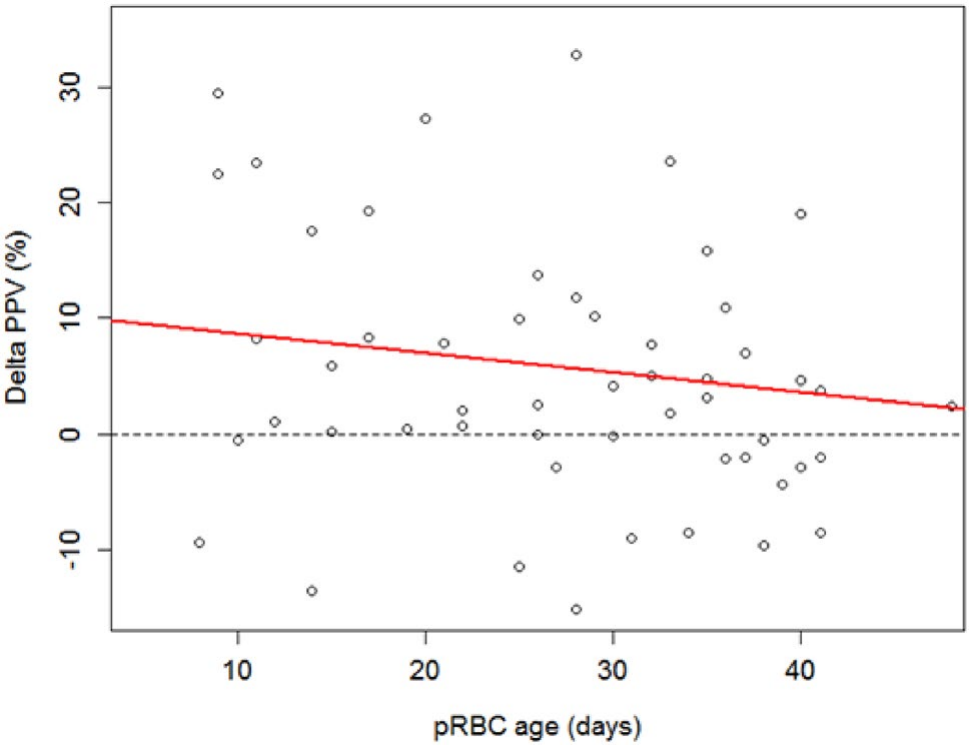
Linear regression model of pre-to post-transfusion proportion of perfused vessels (∆PPV) in percent and the age of pRBC (in days). The red line indicates the trend to a lower increase in ∆PPV in older blood cell concentrates

Linear regression model of ∆PPV in percent and the age of pRBC (in days) is shown in Fig. 1. Changes in PPV were lower in patients who were administered older pRBC. As shown in the regression analysis, no significant effect was observed between *∆*PPV and age of pRBC.

**Fig. 2:**
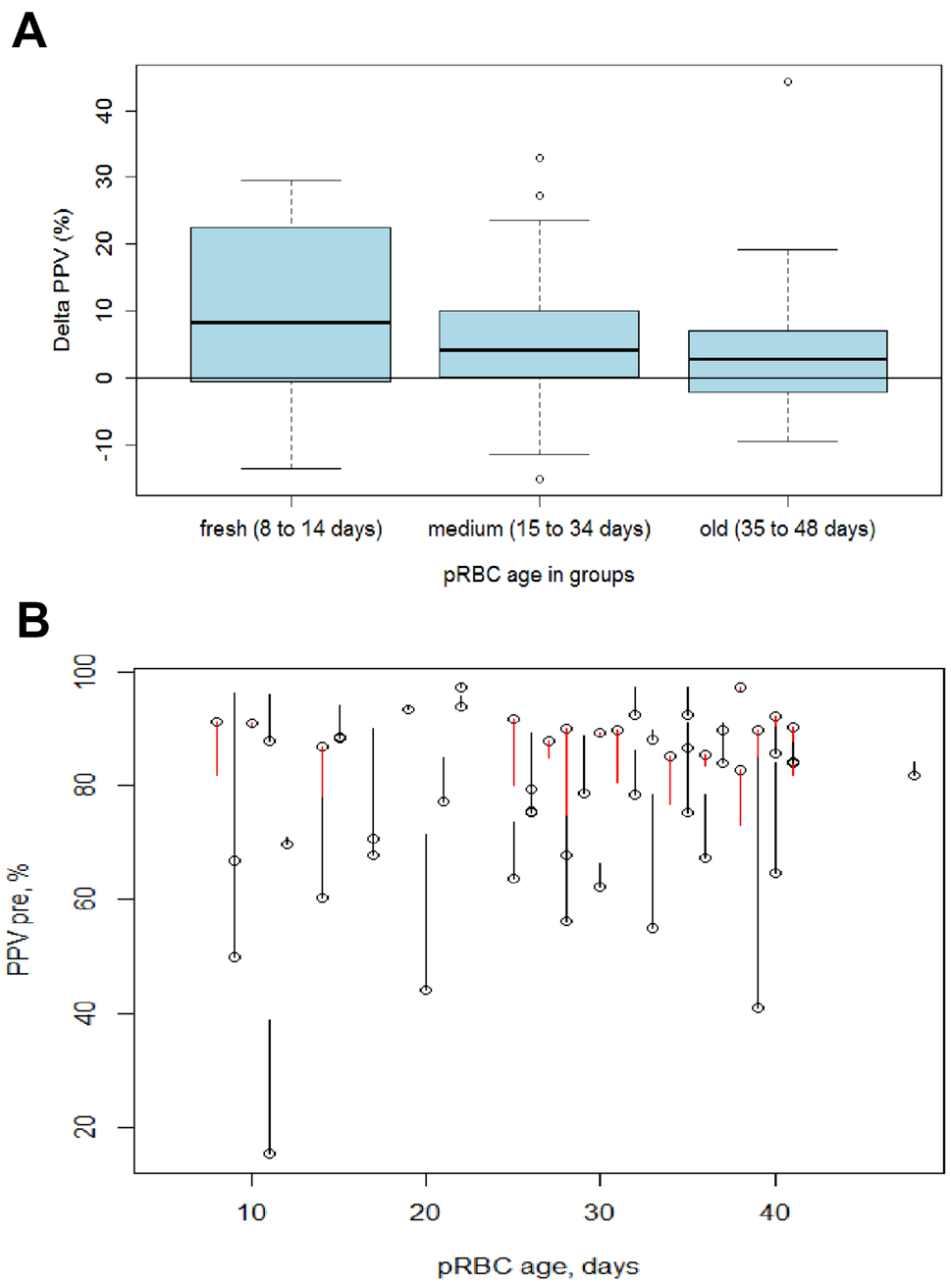
**A** ∆PPV in percent after transfusion of either fresh (< 14 days old, *n* = 9), medium (*n* = 27) or relatively old blood (> 35 days old, *n* = 18). **B** Correlation of pre-transfusion PPV values and the age of the blood product. The dots indicate the pre-transfusion PPV, the lines the development to post-transfusion. Red lines indicate measurements with a lower, black lines with a higher post-transfusion value. Values in the higher pre PPV range are more likely to develop to a negative ∆PPV

Figure 2A shows a boxplot for ∆PPV by pRBC age group with similar values regarding age of transfused pRBC. Patients with a high pre-transfusion PPV level were overrepresented by chance in the older pRBC age group. Therefore, it is less likely that the PPV level truly increases after administration of pRBC (Fig. 2B).

**Fig. 3:**
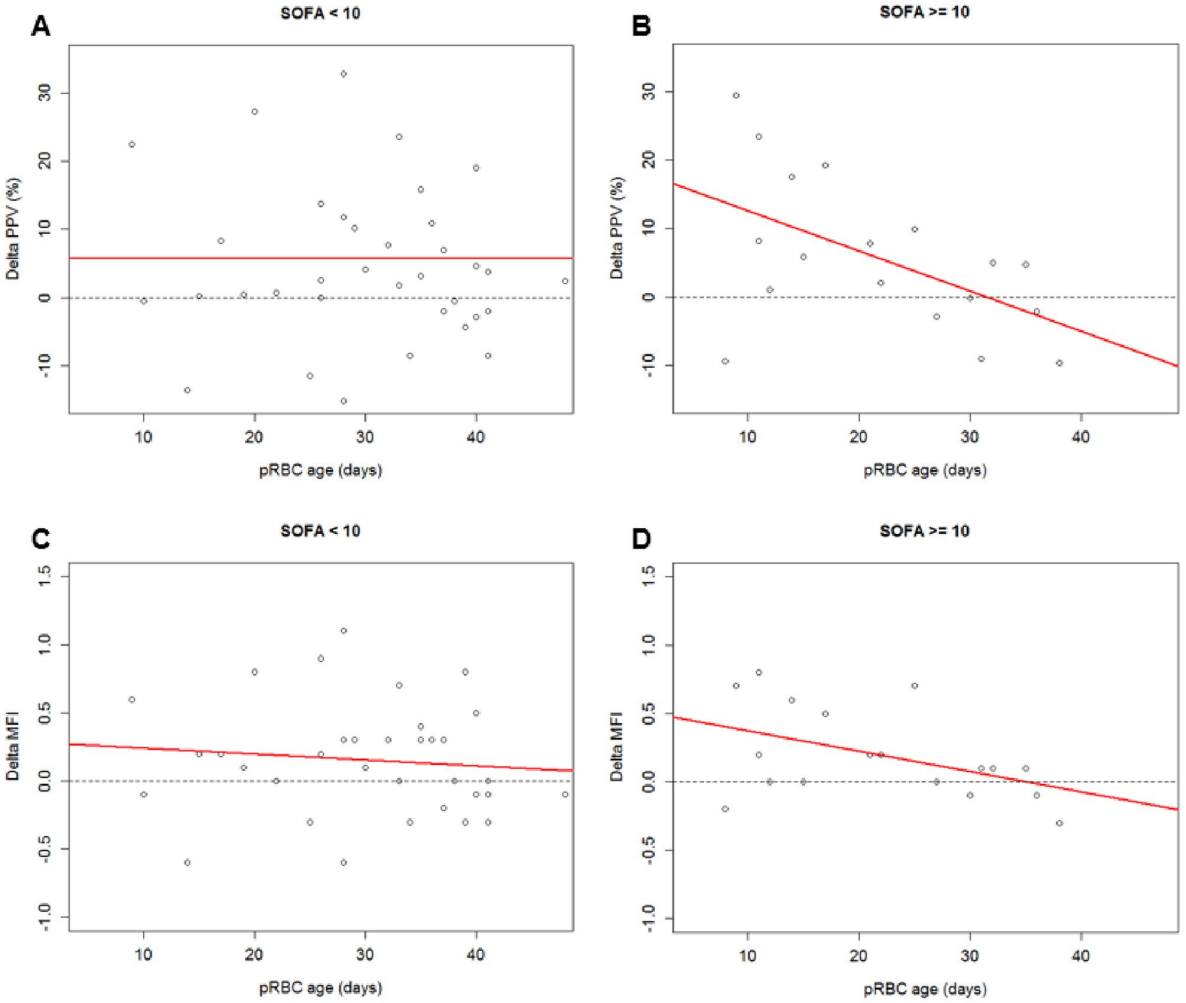
Linear regression model considering ∆PPV (**A**, **B**) or ∆MFI (**C**, **D**) and the age of pRBC in low or high SOFA scores

SOFA score was pathologic (≥ 2 points) in 46 cases (median = 7). With the threshold of ten points for severe cases, 18 patients (33.3%) were grouped above and 36 (66.7%) below the limit. Table 3 shows the results of the regression analysis including co-variables PPV, age of pRBC, the SOFA score as well as an interaction between these two variables (*p* = 0.01). Analogue calculations for perfused vessel density (PVD) and total vessel density (TVD) were made with p-value being < 0.01 and 0.12 respectively (see Appendix Tables 6 and 7). Figure 3 shows the results of this linear regression model for patients with low and high SOFA score for the outcome ∆PPV and ∆MFI. Although no difference can be detected in patients with low SOFA score, there is a trend to lower ∆PPV and ∆MFI in critically ill patients receiving older pRBC.

**Table 3:** Results of regression model, including the interaction of the co-variable SOFA score

Co-variable	Estimate	Confidence interval	p-value
Intercept ∆PPV	− 2.15	(− 19.6 to 15.3)	0.81
Age of pRBC	0.41	(− 0.16 to 0.98)	0.16
SOFA score, high	1.89	(− 0.06 to 3.85)	0.06
Interaction SOFA score and age of pRBC	− 0.09	(− 0.16 to 0.02)	0.01

Adjusted R^2^: 11.2%; *n* = 54

*SOFA* Sequential Organ Failure Assessment; *∆* difference between pre- and post-transfusion measurement; *PPV* proportion of perfused vessels; *pRBC* packed red blood cells

Values for ∆PPV and ∆MFI regarding the grouping with SOFA score and blood age are listed in Table 4.

**Table 4:** Age of pRBC and effects on MCF regarding SOFA score and blood-age groups

Age group	SOFA-score < 10			SOFA-score ≥ 10		
	Fresh	Medium	Old	Fresh	Medium	Old
*n* (of total 54)	3	18	15	6	9	3
Age of pRBC (days)	10 (9.5 to 12)	27 (22.8 to 29.8)	39 (37 to 40.5)	11 (9.5 to 11.8)	25 (21 to 30)	36 (35.5 to 37)
∆PPV (%)	− 0.5 (− 7.1 to 11)	3.3 (0.3 to 11.4)	3.1 (− 2 to 9)	12.9 (2.9 to 22)	5 (− 0.2 to 7.8)	− 2.1 (− 5.9 to 1.4)
∆MFI (SD)	− 0.03 (0.6)	0.23 (0.4)	0.09 (0.3)	0.35 (0.4)	0.19 (0.3)	− 0.1 (0.3)

Fresh (< 14 days old), medium (15 to 34 days old) or relatively old blood (> 35 days old))

*SOFA* Sequential Organ Failure Assessment; *∆* difference between pre- and post-transfusion measurement; *PPV* proportion of perfused vessels; *MFI* microvascular flow index; *pRBC* packed red blood cells; *SD* standard deviation

The relationship between donor’s sex and ∆PPV and ∆MFI (see Appendix Table 8) was not significant (for ∆PPV, *p* = 0.7; for ∆MFI, *p* = 0.3). In addition, no correlation was seen when compared to donor-recipient sex-mismatch. A mismatch was detected in 26 cases (60.47%). Differences between matches and mismatches were not significant for ∆PPV (*p* = 0.5) or for ∆MFI (*p* = 0.6).

## Discussion

Patients receiving blood aged 35 to 48 days did not have a significantly lower ∆PPV after transfusion of pRBC compared to fresher blood. However, in severely critically ill patients (i.e., SOFA score ≥ 10) PPV increased less when older versus fresh pRBC was transfused. In this group of patients, age of pRBC may be of importance, whereby severely ill ICU patients with poor MCF may profit from shorter stored blood. Similarly, calculations for PVD showed these results in the regression model. Two possible explanations could be (1) the pre-existing impairment of the patient’s own erythrocytes due to critical illness, and (2) impaired erythrocyte-to-capillary interaction [19]. Therefore, administered pRBC containing storage lesions might not enter the already altered microcirculation and might not bring this group of patients the same positive effects as fresh pRBC. These findings jeopardize today’s arbitrary haemoglobin-derived transfusion thresholds where neither MCF impairment or severity of illness nor the age of the transfused blood is taken into account.

Microcirculatory blood vessels (i.e., arterioles, capillaries, and venules) are those vessels with a diameter be < 100 μm. Homogenous MCF is crucial for tissue oxygenation [20]. Thus, understanding the role of MCF and implementation of corresponding measurements and study results in the treatment of patients is of great importance but confirmatory data from prospective trials are lacking. In septic patients, for example, persistent MCF alteration correlates with adverse outcome [12, 21–24], thus, warranting treatment [20].

For tissue and microcirculatory perfusion, blood flow is of greater importance than blood pressure [25]. In a prospective study of 20 septic shock patients, MCF alterations could not be improved after elevation of mean arterial pressure (MAP) with norepinephrine [26]. In the second consensus on the assessment of sublingual microcirculation [27], the authors listed heterogeneous blood flow, haemodilution, and stagnant microcirculatory flow due to arterial vasoconstriction or oedema with prolonged oxygen diffusion distances as reasons for discrepant macro- and microcirculatory flow patterns.

Three previous studies examining the relationship between the age of pRBC and the MCF produced divergent findings. Weinberg and colleagues reported decreased perfused capillary density (PCD) after transfusion of older pRBC with consecutive changes in regional microvascular perfusion in a cohort of 93 patients [28]. In contrast, Yürük and colleagues detected no such effects in their 20 patient cohort [29], thereby, concluding that although the impact of storage lesions on haemorheology is well-known, but its clinical relevance remains unclear. Also, Sakr et al. didn’t detect an significant effect of the age of transfused blood on the microvascular perfusion in 35 septic patients receiving pRBC [30].

While the average age of pRBC at the time of transfusion is between 16 and 21 days [1, 31], many countries allow a storage time for up to 42 days [32]. Of note, a decrease in oxygen-delivering capacity was seen after storage of five to six weeks (35–42 days) [7].

With a median storage age of 28.5 days, age of pRBC used in our study was even higher than both our hospital average (mean = 22.9 days) and the international average described above. In order not to waste any blood products, it is a common practice to transfuse the oldest available pRBC first with a minimum time to process a unit of pRBC being 2 days [18].

Overall, there is a slight tendency to a less pronounced increase of blood flow in the microcirculation after transfusion of older blood. However, our data show no statistical significance in the correlation between the age of pRBC up to a maximum storage of 49 days and ∆MCF values. Unequal distribution of data may explain the difference to the previously mentioned inverse correlation with the SOFA subgroups. Moreover, no indication bias was produced as the blood bank always hands out the “oldest” pRBC available and suitable.

Several authors have reported no difference in MCF in studies comparing transfusion of younger (7–20 days old) to older (21 to 42 days old) pRBC [4, 14–16, 29, 33]. Our own comparisons of fresh, medium, and old blood also did not detect a significant difference between MCF and pRBC age. However, these numbers do not respect severity of illness.

A more clinical approach was used in several large randomized controlled trials. Patient outcome, such as mortality, was investigated in the ABLE [34], RECESS [35] or TRANSFUSE [36]. Results of these studies as well as of other trials were summarized in a Cochrane analysis in 2018 [37], and revealed no clear difference in the risk of death after transfusion of blood closer to the expiration date in adults. Based on these findings, transfusion of relatively old blood was considered safe [37]. Nevertheless, these studies also failed to consider the severity of illness. Moreover, mass transfusions were not examined. Studies on the infusion of large volumes of pRBC are thus necessary to shed light on the relevance of storage lesions [38].

Mismatch in sex of donor and recipient of pRBC is described in the literature as a possible factor influencing the outcome of transfusion [39]. We could not detect a benefit in matching pRBC to the recipient’s sex in terms of MCF and also did not find a negative impact of mismatch on flow in capillaries. In addition, no significant differences of donor’s sex and change in MCF could be detected, possibly also due to the small sample size of our study.

Finally, so-called storage lesions due to prolonged storage are well described and understood in in-vitro as well as in in-vivo animal models, but the clinical significance remains unclear [7, 29, 40, 41]. Therefore, the findings of this investigation can only be seen as a jigsaw piece in the field of microcirculation studies with considerable uncertainty. The growing importance of individualized patient treatment in medicine supports bedside measurement of perfusion within the smallest vessels in real-time (MCF), thus serving as an indirect predictor of erythrocyte flow and oxygen delivery to the organs [27, 42].

Our study has several limitations. First, as a retrospective analysis, collection of data about transfused pRBCs was difficult due to missing values in patient charts. Second, the sample size was not powered for the presented research question, and data were not distributed equally due to the standard practice of blood banks to transfuse older pRBCs first. This explains the underrepresentation of fresh blood in this trial. Third, variability of measurements was high: The recording may be facile for an experienced researcher, but to get high quality images may still be challenging. However, the bedside detection of the microcirculation characteristics is far from being simple since it is only qualitative, while the remote manual analysis is affected by important limitations (e.g., analysis variability among centers and operators, lack of an appropriate flow parameter since MFI rather represents a weak parameter for measuring flow). Here, an automated software analysis such as MicroTools,^1^ a validated automatic software freely available for research purposes, could help in the future [43]. In addition, distinctions between different groups may be larger with transfusion of fresh blood being even younger than 8 days.

## Conclusion

Our data support findings that transfusion of older blood, up to 42 days old, does not seem to affect microcirculatory flow. This is in accordance with existing research about microcirculatory changes of blood transfusion and large randomized trials on the outcome of blood transfusions. The trend to lower PPV and MFI in patients with SOFA score > 10 suggest further research on the influence of pRBC transfusions age on the microcirculation in dependence of critical illness severity. Furthermore, we suggest the use of regular measurements of microcirculatory parameters (e.g. capillary refill time or MCF) as a promising tool to be used in the future to adapt transfusions to individual patient requirements rather than simply following arbitrary thresholds.

## Data Availability

The data that support the findings of this study are available from the corresponding author upon reasonable request.

## Appendix

See Tables 5, 6, 7 and 8.

**Table 5:** Transfusion figures

Characteristic	(*n* = 54)	(*n* = 43)
Blood type A, *n* (%)	26 (48.1)	
Blood type B, *n* (%)	6 (11.1)	
Blood type AB, *n* (%)	2 (3.7)	
Blood type 0, *n* (%)	20 (37)	
Rhesus positive, *n* (%)	41 (75.9)	
Age of pRBC at transfusion, (days; median and IQR)	28.5 (19.3–36)	
Donor sex, male, *n* (%)		27 (62.8)
Donor–recipient sex-match, *n* (%)		17 (39.5)
Donor–recipient sex-mismatch, *n* (%)		26 (60.5)

*IQR* interquartile range; *pRBC* packed red blood cells

**Table 6:** Regression model with ∆PVD, including the interaction of the co-variable SOFA score

Co-variable	Estimate	Confidence interval	p-value
Intercept ∆PVD	5.08	(0.63 to 9.55)	0.03
Age of pRBC	− 0.19	(− 0.33 to − 0.04)	0.01
SOFA score, high	− 1.12	(− 1.62 to − 0.62)	< 0.01
Interaction SOFA score and age of pRBC	0.04	(0.02 to 0.05)	< 0.01

Adjusted R^2^: 29.1%; *n* = 54

*SOFA* Sequential Organ Failure Assessment; *∆* difference between pre- and post-transfusion measurement; *PVD* perfused vessel density; *pRBC* packed red blood cells

**Table 7:** Regression model with ∆TVD, including the interaction of the co-variable SOFA score

Co-variable	Estimate	Confidence interval	p-value
Intercept ∆TVD	5.98	(0.87 to 11.08)	0.02
Age of pRBC	− 0.18	(− 0.35 to − 0.01)	0.04
SOFA score, high	− 0.60	(− 1.17 to − 0.3)	0.04
Interaction SOFA score and age of pRBC	0.02	(− 0.00 to 0.04)	0.12

Adjusted R-squared: 6.2%; *n* = 54

*SOFA* Sequential Organ Failure Assessment; *∆* difference between pre- and post-transfusion measurement; *TVD* total vessel density; *pRBC* packed red blood cells

**Table 8:** pRBC and effects on microcirculatory flow regarding sex and donor-recipient sex-mismatch

	All	Sex of donor		Donor-recipient sex-mismatch	
		Male	Female	Match	Mismatch
*n*	43	27	16	17	26
Age of pRBC (days)	28.0 (17 to 36.5)	28.0 (16 to 36.5)	27.5 (18.5 to 35.8)	32.0 (25 to 37)	26.0 (17 to 35.8)
∆PPV (%)	3.8 (− 2 to 10.4)	2.0 (− 2.1 to 9.1)	4.9 (− 0.1 to 13.2)	1.8 (− 2.9 to 9.9)	4.0 (− 5 to 10.9)
∆MFI (SD)	0.15 (0.4)	0.11 (0.4)	0.23 (0.3)	0.12 (0.4)	0.17 (0.3)

*∆*Difference between pre- and post-transfusion measurement; *PPV* proportion of perfused vessels; *MFI* microvascular flow I; *pRBC* packed red blood cells; *SD* standard deviation

## Abbreviations

Ba^2^MiTraL: Basel Bedside assessment Microcirculation Transfusion Limit study
CI: Confidence interval
∆: Delta
ICU: Intensive care unit
Hb: Haemoglobin
MCF: Microcirculatory flow
MFI: Microvascular flow index
PAGGS-M: Phosphate-adenine-glucose-guanosine-saline-mannitol
PPV: Proportion of perfused vessels
pRBC: Packed red blood cells
PVD: Perfused vessel density
SAGM: Saline-adenine-glucose-mannitol
SD: Standard deviation
SOFA: Sequential Organ Failure Assessment
TVD: Total vessel density

## Footnotes

1 http://www.nature.com/articles/s42003-019-0473-8
